# Rating Hospital Performance in China: Review of Publicly Available Measures and Development of a Ranking System

**DOI:** 10.2196/17095

**Published:** 2021-06-17

**Authors:** Shengjie Dong, Ross Millar, Chenshu Shi, Minye Dong, Yuyin Xiao, Jie Shen, Guohong Li

**Affiliations:** 1 School of Public Health Shanghai Jiao Tong University School of Medicine Shanghai China; 2 Health Services Management Centre University of Birmingham Birmingham United Kingdom; 3 Center for Health Technology Assessment China Hospital Development Institute Shanghai Jiao Tong University Shanghai China; 4 China Hospital Development Institute Shanghai Jiao Tong University School of Medicine Shanghai China

**Keywords:** hospital ranking, performance measurement, health care quality, China health care reform

## Abstract

**Background:**

In China, significant emphasis and investment in health care reform since 2009 has brought with it increasing scrutiny of its public hospitals. Calls for greater accountability in the quality of hospital care have led to increasing attention toward performance measurement and the development of hospital ratings. Despite such interest, there has yet to be a comprehensive analysis of what performance information is publicly available to understand the performance of hospitals in China.

**Objective:**

This study aims to review the publicly available performance information about hospitals in China to assess options for ranking hospital performance.

**Methods:**

A review was undertaken to identify performance measures based on publicly available data. Following several rounds of expert consultation regarding the utility of these measures, we clustered the available options into three key areas: research and development, academic reputation, and quality and safety. Following the identification and clustering of the available performance measures, we set out to translate these into a practical performance ranking system to assess variation in hospital performance.

**Results:**

A new hospital ranking system termed the China Hospital Development Index (CHDI) is thus presented. Furthermore, we used CHDI for ranking well-known tertiary hospitals in China.

**Conclusions:**

Despite notable limitations, our assessment of available measures and the development of a new ranking system break new ground in understanding hospital performance in China. In doing so, CHDI has the potential to contribute to wider discussions and debates about assessing hospital performance across global health care systems.

## Introduction

Hospital rating systems have the potential to play an important role in patient decision-making as well as offer policy makers and practitioners valuable opportunities to monitor and improve the quality of hospital services [1-4]. In China, significant emphasis and investment into health care reform since 2009 has brought with it increasing scrutiny of its public hospitals with regard to improving their quality and efficiency. Reform measures have included an emphasis on improving hospital governance with clearer regulations and transparency regarding overall performance [5]. Although these measures show promising signs, questions remain about their overall impact and sustainability [6], as well as those concerning the information asymmetries that exist for patients and providers that limit the market conditions of competition and choice deemed necessary to rate hospital performance [7].

An enduring feature of China’s health care provision is the dominance of the hospital sector. Within these contexts, patients are offered different forms of provision ranging from grade I community hospitals, grade II secondary or county hospitals serving several communities, and grade III tertiary hospitals serving districts or cities. This classification [8] remains a powerful driving force for decision-making, with tertiary hospitals often deemed the preferred option for better clinical quality. Pan et al [7] explain how such trends are driven by a culture where patient volume often represents the primary measure of hospital performance used by government administrators. Patients often equate hospital size as a signal of quality, thus preferring to self-refer to larger tertiary hospitals. Large patient volume is also deemed essential for hospitals in developing a good reputation and acquiring high-quality research and training programs.

U.S. News & World Report’s Best Hospitals ranking is one of the well-known hospitals ranking systems that aims to help patients find professional medical centers and doctors across the United States. The relative success of the Best Hospitals ranking demonstrates that the objectivity of measures such as mortality and morbidity can provide an important contribution for accurate evaluation of health care quality [9]. However, influential rankings in developed countries, such as Best Hospitals ranking and Vizient Award [10], are based on solid medical information supporting mechanisms and are challenging to be applied to low- and middle-income countries or regions with relatively underdeveloped medical information supporting facilities.

In order to try and disentangle these trends, China is increasingly seeing the development and use of hospital performance rankings. The annual publication of the Hospital Management Institute of Fudan University Hospital Ranking list [11] ranks hospitals according to a social reputation score that is determined based on survey responses from physicians combined with a review of scientific research outputs from their affiliated institutions. The Science and Technology Evaluation Metrics (STEM) of hospitals developed by the Chinese Academy of Medical Sciences [12] ranks tertiary hospital performance based on their science and technology investment and any associated outputs. Other influential rankings include the top-100 China Hospitals Competitiveness by Alibi Hospital Management Research Center, Hong Kong [13], and China’s best clinical discipline rankings released by Peking University [14].

These indicators provide a valuable contribution to debates and decision-making about hospital performance in China. Nevertheless, given the current situation in China, and the asymmetries of information that exist, important limitations of the current ranking systems have been highlighted, including a reliance on reputation scores [11], a limited menu of performance measures, and a lack of consideration and engagement with measures of quality and safety [8].

Current ranking systems undoubtedly have merit in signifying attempts to better understand hospital performance; however, further research is needed to better understand and triangulate publicly available hospital performance information. Thus far, there has yet to be a comprehensive analysis of what performance information is actually available in China [15]. This study aims to review the publicly available performance information with the view to assess different ways in which hospital performance can be ranked. In doing so, in this paper, we present a new hospital ranking system, termed the “China Hospital Development Index” (CHDI). Although this ranking system faces notable limitations, we argue that our review of measures and development of a ranking system break new ground that can inform both current and future policy and practice for hospital performance in China.

## Methods

### Limitations of Major Hospital Ranking Systems in China

Current hospital performance rankings in China [11-13] classify hospital performance across a range of indictors, including the availability of hospital facilities, services, and personnel; the calculation of social reputation scores; and the publication of scientific research inputs and outputs. These indicators provide valuable contributions for understanding hospital performance; however, a notable limitation in the rankings produced so far has been the emphasis on the quality and safety of health care provision. Quality and safety represent core domains of medical services; therefore, any assessment of hospital performance should aim to incorporate any available measures [16].

It is worth pointing out that for the clinical disciplines ranking reported in Table 1, more than 48 million clinical data records were collected from nearly 400 hospitals across China from 2006 to 2014. The main characteristic of this ranking is that the focus has been shifted to the clinical specialties rather than the number of funds and articles published; it is also the first application of effective medical clinical data for evaluation of hospitals. It should be mentioned that its methodology has not been made public. However, this ranking was unsuccessful (published only once in 2015). The main reason is the standardization of clinical data, such as inconsistent disease codes, which directly affects the quality of medical record information used. Although hospitals in China are vigorously promoting medical informatization at this stage, there is still a long way to use medical data to rank hospitals even if such data are available.

**Table 1:** Overview of China’s hospital ranking systems.

Characteristic	Best Hospitals ranking [11]	STEM^a^ [12]	Hospital competitiveness ranking [13]	Best Clinical Disciplines ranking [14]
Primary objective	To provide guidelines for patients seeking treatments	To measure a hospital’s value in scientific research	To identify the top hospitals with the best competitiveness	To provide a tool to help patients find skilled specialty care
Domains of measure	Social reputation The ability of sustainable development (scientific research outcomes)	Scientific research inputs, outputs, and impacts	Medical service Academic impacts Resource management Hospital operation	Unclear
Indicators	Social reputation scores SCI^b^ papers National awards	Key laboratories and projects Researchers Clinical trials SCI papers Medical standards Medical association leaders National awards	Inpatients and outpatients Beds Health workers Medical facilities Personnel Medical fee Length of stay Academic leaders Key laboratories and projects National awards	Perioperative mortalities Readmissions Postoperative complications Timely care Finance
Data sources	National surveys	SCI database and official documents	Reporting data voluntarily	Medical records
Publication frequency	Annually	Annually	Annually	Only in 2015
Transparency of methodology	Provided	Provided	Provided	Not provided

^a^STEM: Science and Technology Evaluation Metrics.

^b^SCI: Science Citation Index.

### Exploring Available Performance Measures

Based on the assessment of current measures and the identification of areas for improvement, a group of 6 experts with physician and methodological expertise in performance measurement was established within the China Hospital Development Institution (HDI) to assess the available options and provide feedback at each step of the process (see Multimedia Appendix 1). To begin the analysis, we mapped publicly available measures and identified different information sources that reflected our interest in better understanding *hospital performance*. Through iterative discussions among the study group, a review of the available literature, and discussions with experts, we established three performance domains for the purpose of ranking hospitals in China. These domains were categorized as research and development, academic reputation, and quality and safety (described below and summarized in Table 2).

**Table 2:** Summary of measures and clustering of performance indicators into three domains: research and development, academic reputation, and quality and safety.

Domains and measures			Indicators		Data sources
**Research and development**					
	Publication outputs	Number of SCI^a^ papers by authors, by first author, and by corresponding author		Web of Science [17]	
	Number of citations	Number of citations by authors, by first author, and by corresponding author		Web of Science [17]	
	Number of high-impact outputs	Number of SCI papers with journal IF^b^ ≥10 by authors, by first author, and by corresponding author; number of papers published in top-6 journals by authors, by first author, and by corresponding author		Web of Science [17]	
	Clinical trial activity	Number of registered clinical trials		ChiCTR^c^ [18]	
**Academic reputation**					
	Academician	Number of academicians of CAS^d^ and CAE^e^		CAS [19], CAE [20]	
	Chief editor	Number of staff as chief editors of core medical journals included in CSCD^f^		CSCD, Science China [21]	
	Association chairperson or member	Number of staff as National Association chairperson and National Association members		CMA^g^ [22], CMDA^h^ [23]	
	Award	SPSTA^i^, SNSA^j^, STTPA^k^, CMSTA^l^, CDA^m^		CMA [22], CMDA [23], NOSTA^n^ [24]	
**Quality and Safety**					
	Quality of specialty care	Number of national key clinical specialties, diagnosis and treatment, Improvement of Rare Diseases Program		Official websites of each hospital; NHC^o^, People’s Republic of China [25]	
	Medical malpractice claims	Ratio of compensation cases, ratio of liability		Laws and Regulations – Peking University [26]	

^a^SCI: Science Citation Index.

^b^IF: impact factor.

^c^ChiCTR: Chinese Clinical Trial Registry.

^d^CAS: Chinese Academy of Sciences.

^e^CAE: Chinese Academy of Engineering.

^f^CSCD: China Science Citation Database.

^g^CMA: Chinese Medical Association.

^h^CMDA: Chinese Medical Doctor Association.

^i^SPSTA: State Preeminent Science and Technology Award.

^j^SNSA: State Natural Science Award.

^k^STTPA: State Scientific and Technological Progress Award.

^l^CMSTA: Chinese Medical Science and Technology Award.

^m^CDA: Chinese Doctor Award.

^n^NOSTA: National Office for Science and Technology Awards

^o^NHC: National Health Commission.

To begin our analysis of available measures, we used the Health Statistics Yearbook issued by the National Health Committee to gather baseline information regarding outpatient, inpatient, and emergency admissions to hospitals in China [27]. Second, given the importance placed on research and development in China as a measure of performance, we also sought to identify research and development indicators for hospitals gathered from research databases in order to gauge the research activity and outputs being produced by each hospital. This would include any hospital affiliation of authorship to published Science Citation Index (SCI) papers, the number of citations obtained, and the number of SCI papers for which an impact factor (IF) ≥10 per hospital. Information regarding clinical trial activity was also collected as an indicator for research activity.

As a follow-up to the research and development activity, we were able to obtain measures demonstrating clinical academic reputation of hospital staff engaged in high-impact research outputs demonstrating wide scholarly impact in their clinical area of expertise. This included measuring the number of academic affiliations with the Chinese Academy of Science (CAS) and the Chinese Academy of Engineering (CAE); the number of staff as chief editors of core medical journals included in the China Science Citation Database (CSCD); membership of national associations, including the Chinese Medical Association (CMA) and the Chinese Medical Doctor Association (CMDA); and the number of national awards received per hospital, including the State Preeminent Science and Technology Award (SPSTA), the State Natural Science Award (SNSA), the State Scientific and Technological Progress Award (STTPA), the Chinese Medical Science and Technology Award (CMSTA), and the Chinese Doctor Award (CDA).

Finally, our analysis of the quality and safety performance measures was able to draw on medical malpractice litigation records [28,29] adjusted for complexity and risk of patient disease as two useful indicators for patient safety. Based on the experience the team had in analyzing litigation data as a measure of quality, the selection of such measures resonates with others such as Wang et al [28], who argued that in the absence of more robust indicators, records of medical malpractice litigation in China warranted further exploration as an indicator of health care quality. Additional measures of clinical quality were accessed by reviewing hospital standards and accreditation of treatment excellence performance against the National Health Commission’s Diagnosis and Treatment Improvement of Rare Diseases Program and National Key Clinical Specialty Program.

### Developing a Hospital Ranking System

Several rounds of expert consultation identified publicly available indicators, deliberated their utility, and assessed how best to triangulate and weight these measures into comparative performance information. Following the identification and clustering of the available performance measures, we set out to translate these into a practical performance ranking table to assess variation in hospital performance.

Our analysis of operational size and scale highlighted practical limitations to the sample of hospitals included in our ranking. As a result, we focused on the tertiary hospital sector based on the availability of current data as well as to provide an option for comparison with other available measures. By the end of 2017, according to the China Health Statistics Yearbook, there were 1360 grade III, level A hospitals nationwide [27]. The inclusion of these hospitals over others was on the basis that these organizations continue to be the focus of attention in China given their prominence and popularity. These hospitals have also been the focus on other performance rankings in China; hence, the development of any new ranking system would be comparable with other respective performance measures. Our inclusion criteria, therefore, required hospitals to be a grade III, level A hospital, featuring on one of the lists of the four Chinese Hospital rankings in any previous year, and have at least 500 beds. A total of 310 hospitals were thus deemed eligible for ranking under the full criteria.

To develop our ranking system, we relied on statistical procedures such as principal component analysis (PCA) and categorical principal component analysis (CATPCA) [30,31]. PCA is defined as a variable reduction technique that can be used when variables are highly interrelated, providing a way to reduce the number of observed variables into a smaller number of linear, uncorrelated summary variables called principal components (PCs) that account for variation in observed variables. Here, we hypothesized that the various candidate indicators for a given hospital can be represented by several underlying, or latent PCs that reflect the overall strength of this hospital. Thus, for each PC, the model can estimate the extent to which the values are the result of a relationship with the composite score. The remaining variance in the indicators is attributed to measurement error. The degree to which an indicator is correlated with other indicators helps to determine its weight in the equation for the composite scores.

We developed PCA/CATPCA models for each of the three domains of ranking, by evaluating model statistics for all possible combinations of indicators that included at least one indicator. From the resulting list of candidate models showing acceptable fit statistics, we selected a final model for each domain, providing a combination of the number of indicators (models with more indicators produce more accurate component scores), number of outcomes, and model fit.

The PCA/CATPCA replaces the original *n* features with a smaller number of *m* features, which are linear combinations of the old features, making these linear combinations as irrelevant as possible. To adjust for linear distribution, before incorporating the values of PCs into the scoring, we implemented a logit transformation for each domain. Equation (1) presents the formula for logit transformation:


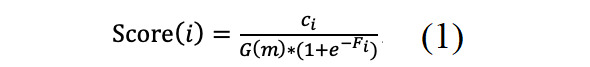


Score(*i*): final score for PC*_i_*; *i*: PC*_i_*; G(*m*): accumulation of variance; C*_i_*: variance for PC*_i_*; and F*_i_*: original score for PC*_i_*.

### Entropy Weight Method

For each domain, we incorporated the values of PCs into the scoring by using entropy weight method, in which weights are systematically calculated based on the level of the difference between the original values. Simply put, if the value difference between the objects, when evaluated using an indicator, is higher than the difference using other indicators, that indicator has more weight than other indicators [32].

Matrix after logical transformation:


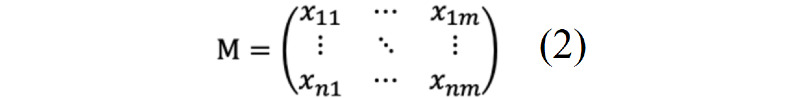


where *n*: number of hospitals; *m*: number of variables.


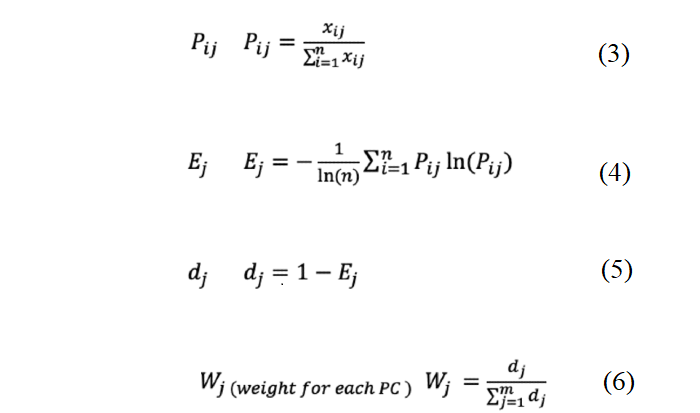


### Weighting

Deliberations between the expert panel of HDI stakeholders determined the appropriate weights for each domain based on their importance in defining the overall attributes of strength within hospitals.

For presentation purposes, we created what we define as CHDI to measure the development level of the hospitals evaluated. Raw scores were transformed to a scale that assigns a CHDI score of 100 to the top hospital. The formula for the transformation is shown in Equation (7):


CHDI score = (raw score – minimum) / range (7)

Before applying PCA, we also measured the correlation of each variable using Kaiser–Meyer–Olkin (KMO) analysis. If the KMO value is >.7, there is a relatively high level of correlation among variables, and it is thus suitable to use PCA. Similarly, we calculated Cronbach α coefficient before applying CATPCA. CATPCA is an alternative to standard PCA that is particularly useful for data sets consisting of categorical variables (nominal or ordinal) that might be nonlinearly related to each other. CATPCA quantifies categorical variables using optimal scaling, resulting in optimal PCs for the transformed variables. The correlations, shown in Table 3, provide strong evidence of construct validity.

**Table 3:** Kaiser–Meyer–Olkin (KMO) analysis (Cronbach alpha values) of the three domains of the China Hospital Development Index.

Domain	KMO/Cronbach α
Research and development	.850
Academic reputation	.802
Quality and safety	.936

## Results

### PCA Results

Table 4 shows the results of the analysis, including the number of original indicators, number of selected indicators, number of PCs retained, and accumulation of variance for each domain. The PCA resulted in two PCs, which explained no less than 81% of the variance in the original matrix for each domain.

For research and development, the first principal component (PC1) is highly correlated with the amount of SCI papers and citations, whereas the second principal component (PC2) is highly correlated with high-quality paper measures such as “number of IF≥10 SCI papers by authors” and “number of IF≥10 SCI papers by first author” with correlation coefficients of 0.489 and 0.575, respectively, between the original variables and PCs identified. For quality and safety, PC1 is highly correlated with the medical malpractice claims measures, whereas PC2 is highly correlated with the quality of specialty care measures.

**Table 4:** Principal component analysis and categorical principal component analysis results of the three domains of the China Hospital Development Index.

Domain	Original indicators	Selected indicators	Principal component	Accumulation of variance
Research and development	13	11	2	0.936
Academic reputation	9	6	2	0.818
Quality and safety	3	3	2	0.887

### Results of Entropy Weight Method

Table 5 shows PC1 has a higher entropy weight than PC2 for each domain, suggesting that this component has a bigger difference.

The results of our CHDI rankings by score for the top 10 hospitals are shown in Table 6.

**Table 5:** Results of the entropy weight method for the three domains of the China Hospital Development Index.

Domain	PC1^a^ (%)	PC2^b^ (%)
Research and development	85.4	14.6
Academic reputation	70.2	29.8
Quality and safety	85.1	14.9

^a^PC1: first principal component.

^b^PC2: second principal component.

**Table 6:** An example of the China Hospital Development Index (CHDI) ranking results.

Rank	Hospital	CHDI
1	XH Hospital, Beijing	0.9739
2	HX Hospital, Chengdu	0.9635
3	JZ Hospital, Beijing	0.9634
4	RJ Hospital, Shanghai	0.9632
5	ZS Hospital, Shanghai	0.9527
6	HS Hospital, Shanghai	0.9400
7	ZY Hospital, Hangzhou	0.9284
8	CH Hospital, Shanghai	0.9206
9	RM Hospital, Beijing	0.9171
10	BY Hospital, Beijing	0.9137

### Validation

Table 7 shows the correlation between scores of domains for 310 hospitals. All of the correlation coefficients between the total score and the score of each domain are above 0.57. The scores of different domains also correlate well among themselves with correlation coefficients higher than 0.50, indicating that the set of indicators is compact and coherent.

**Table 7:** Correlation coefficients (*r*) between scores of the three domains for all hospitals evaluated (N=310).

Domain		Research and development	Academic reputation	Quality and safety	Total score
**Research and development**					
	*r*	1	0.788	0.577	0.959
	*P* value	—^a^	<.001	<.001	<.001
**Academic reputation**					
	*r*	0.788	1	0.597	0.857
	*P* value	<.001	—	<.001	<.001
**Quality and safety**					
	*r*	0.577	0.597	1	0.773
	*P* value	<.001	<.001	—	<.001
**Total score**					
	*r*	0.959	0.857	0.773	1
	*P* value	<.001	<.001	<.001	—

^a^Not applicable.

## Discussion

### Principal Findings

The ability to assess the performance of hospitals in supporting the delivery of high-quality patient care represents a priority for all health care systems [33]. In China, such interest and scrutiny are growing; however, gaining access to relevant performance information remains challenging [34]. Through our analysis of available performance measures, our study aims to contribute to these discussions and debates with a review of hospital performance measures in China. Compared to other health care systems, most notably those in the United States and Europe, what these various measures show are clear limitations in what is currently available to understand hospital performance. For example, our use of research and development indicators and academic reputation as proxy measures for managerial and clinical leadership are exposed to criticisms for their limited connections to day-to-day hospital practice. Our use of litigation data and accreditation standards as proxy measures for hospital quality and safety again have limitations in terms of how far these reflect the clinical quality of hospital care [35]. There is further work to be done regarding how China can develop more clinically focused performance measures that are comparable across hospitals.

Nevertheless, we would argue that our review and subsequent development of a new ranking system breaks new ground in understanding hospital performance in China. Where current hospital rankings in China often rely on reputation scores and investment (input) measures [8,11,12], our review of publicly available measures and their development into a ranking system appears to be the first in opening up the debate for more rigorous and transparent performance information. This is particularly the case for quality and safety performance. Our review and inclusion of litigation data [28,29,36,37] provides a valuable opportunity to assess the comparative performance of quality and safety across hospitals in China.

Thus, this paper contributes to what appears to be a growing body of knowledge that is using innovative and feasible methodologies in data collection and modeling to better understand the performance of public hospitals in China [38].

Based on our analysis, we suggest that further research and policy development is needed to build on these results. Given the practical limitations of securing comparative data and the interest in benchmarking our analysis with existing hospital rankings in China, our sample has focused exclusively on a number of tertiary hospitals. Our rankings reflect the high performance of these organizations compared to that of other hospital and primary care providers; however, we are also mindful of the possible further imbalance this can create in China’s health care system by virtue of acute medical care over primary and community care. The fact that the majority of our highest ranked hospitals are located in Shanghai and Beijing also illustrates important challenges facing access to high-quality hospital care in other parts of China. Such findings support those of Yu et al [38] who have documented how the unevenness of health care resources in China is closely related to a city’s administrative rank and power: the higher the level, the better the resources. Such arrangements are reinforcing investment in high-ranked hospitals at the expense of primary care services. The correlation between the quality and safety domain and the overall hospital performance in our ranking system is slightly lower than that with the other two domains; therefore, more clinical objective measures should be included to increase the influence of this domain.

Therefore, we call on further research and development to access and compare performance measures from within each hospital, including private hospitals, as well as other parts of the health care system, including primary and community care. For this purpose, China could build on the cross-sectional research it has undertaken into mortality trends [39] and nurse staffing levels [40]. Such research has the potential to be scaled up and developed into performance measures translatable across all hospitals and incorporated into our methodology.

We also support further research and development that draws on the views of a range of different stakeholders in terms of what performance measures would be meaningful for patients, public, and health care staff. Given the well-documented challenges facing the doctor-patient relationship in China [41,42], we encourage deliberative events involving a range of stakeholders to discuss what constitutes good performance with the view to developing shared understanding of performance measurement from different perspectives. The Delphi method [43] is one way to do this, and such an approach has been used in other parts of China to good effect. This includes further development of comparative measures for health outcomes and the development of experiential data about how different stakeholders experience the health care received and how they can improve hospital performance as well as other aspects of China’s health care system.

The year 2019 marks the tenth year for China’s goal to deepen the reform of its medical and health system. In 2019, the Chinese government formulated the National Tertiary Public Hospital Performance evaluation index system and unified the collection of performance information across hospitals [44]. The implications of such changes remain to be seen, with the results of these assessments not yet fully disclosed. However, we anticipate this is an important step in developing greater understanding of hospital performance in China. We believe that our review and the newly developed ranking index (CHDI) has an important role to play in shaping such discussions and assessments, particularly in relation to the improvement of quality and patient safety, as well as raising public awareness regarding the information that is available to inform their decision-making.

### Conclusions

The reform of China’s health care system has brought with it increasing scrutiny regarding the quality of care delivered to patients. Our analysis presents what appears to be the first review of publicly available performance measures for hospitals in China. In collaboration with an expert panel, in the review, the available measures have been clustered into three performance domains, namely research and development, academic reputation, and quality and safety of hospital care. Furthermore, based our analysis, we have applied these performance measures to a selection of tertiary hospitals in China with the view to better understand their comparative performance. There remain some notable limitations and challenges regarding this performance information; nevertheless, we believe that our review and ranking system break new ground in assessing hospital performance in China. Although further research and development is clearly needed to enhance and refine this performance information, we argue that the proposed hospital development index sets a new and important research agenda for understanding and improving hospital care in China.

## Appendix

Multimedia Appendix 1 Ranking process of China Hospital Development Index.

## Abbreviations

CAE: Chinese Academy of Engineering
CAS: Chinese Academy of Sciences
CATPCA: categorical principal component analysis
CDA: Chinese Doctor Award
CHDI: China Hospital Development Index
CMA: Chinese Medical Association
CMDA: Chinese Medical Doctor Association
CMSTA: Chinese Medical Science and Technology Award
CSCD: China Science Citation Database
HDI: Hospital Development Institution
IF: impact factor
KMO: Kaiser–Meyer–Olkin
PC: principal component
PC1: first principal component
PC2: second principal component
PCA: principal component analysis
SCI: Science Citation Index
SNSA: State Natural Science Award
SPSTA: State Preeminent Science and Technology Award
STEM: Science and Technology Evaluation Metrics
STTPA: State Scientific and Technological Progress Award
