# Correction: Rating Hospital Performance in China: Review of Publicly Available Measures and Development of a Ranking System

**DOI:** 10.2196/31370

**Published:** 2021-06-22

**Authors:** Shengjie Dong, Ross Millar, Chenshu Shi, Minye Dong, Yuyin Xiao, Jie Shen, Guohong Li

**Affiliations:** 1 School of Public Health Shanghai Jiao Tong University School of Medicine Shanghai China; 2 Health Services Management Centre University of Birmingham Birmingham United Kingdom; 3 Center for Health Technology Assessment China Hospital Development Institute Shanghai Jiao Tong University Shanghai China; 4 China Hospital Development Institute Shanghai Jiao Tong University School of Medicine Shanghai China

In “Rating Hospital Performance in China: Review of Publicly Available Measures and Development of a Ranking System” (J Med Internet Res 2021;23(6):e17095) the authors noted one error.

An author was incorrectly included in the authorship list on the published paper. This author has been removed from the corrected version of the manuscript. The correct author list is Shengjie Dong, Ross Millar, Chenshu Shi, Minye Dong, Yuyin Xiao, Jie Shen, Guohong Li.

The correction will appear in the online version of the paper on the JMIR Publications website on June 22, 2021, together with the publication of this correction notice. Because this was made after submission to PubMed, PubMed Central, and other full-text repositories, the corrected article has also been resubmitted to those repositories.

