# Supplementary material for: Rating Hospital Performance in China: Review of Publicly Available Measures and Development of a Ranking System
Source: J Med Internet Res. 2021 Jun 17;23(6):e17095. doi: 10.2196/17095 (PMC8277410; doi:10.2196/17095)
Supplement: Multimedia Appendix 1 [file jmir_v23i6e17095_app1.docx]

**Process of CHDI Ranking**

**Dissemination of rankings**

- Report
- Conference with local selected hospitals

**Reevaluate each hospital and make the ranking list**

**Validation Meeting**

- Discuss issues identified from the primary results
- Compare with other rankings
- Refine ranking methodology

**Collect data of selected hospitals**

**Data analysis and primary results**

**Further define and refine evaluation domains**

**Evaluation Meetings**

- Identify indicators based on publicly available data
- Discuss ranking methodology

**Preliminary Meetings**

- Identify objectives
- Identify evaluation domains
- Select hospitals to evaluate
- Discuss data sources
